# Fear of Disease Progression in Patients with Alveolar Echinococcosis: Associations with Psychological, Psychosocial, and Medical Factors

**DOI:** 10.3390/pathogens15101077

**Published:** 2026-10-12

**Authors:** Małgorzata Sulima, Agata Zdun-Ryżewska, Magdalena Błażek, Katarzyna Sikorska

**Affiliations:** 1 Division of Tropical and Parasitic Diseases, University Centre of Maritime and Tropical Medicine, Medical University of Gdańsk, 80-210 Gdańsk, Poland; ksikorska@gumed.edu.pl; 2 Division of Quality of Life Research, Department of Psychology, Faculty of Health Sciences with the Institute of Maritime and Tropical Medicine, Medical University of Gdańsk, 80-210 Gdańsk, Poland; magdalena.blazek@gumed.edu.pl; 3 Division of Tropical Medicine and Epidemiology, Institute of Maritime and Tropical Medicine, Faculty of Health Sciences, Medical University of Gdańsk, 80-210 Gdańsk, Poland

**Keywords:** fear of disease progression, alveolar echinococcosis

## Abstract

Introduction: Alveolar echinococcosis is a chronic and potentially severe parasitic disease that often requires long-term treatment and is associated with considerable uncertainty regarding prognosis. These characteristics may contribute to significant psychological burden in affected individuals. The aim of this study was to assess the intensity of fear of disease progression among patients diagnosed with echinococcosis treated with different therapeutic approaches and to identify medical and psychosocial factors associated with higher levels of this fear. Method: A total of 35 patients with a diagnosis of alveolar echinococcosis participated in the study. Self-report questionnaires were used to measure anxiety, stress, and knowledge about the disease. Additional data were collected regarding the course of treatment and the nature of social responses after disclosure of the diagnosis. Results: A high level of fear of progression was reported by many patients—seven individuals rated their fear as maximal or near-maximal (9–10 on a 0–10 scale). Statistical analysis showed no significant associations between fear levels and medical variables such as treatment type, drug importation, or presence of side effects. However, a significant relationship was found between the intensity of fear and social reactions. Patients who experienced non-supportive or stigmatizing responses (e.g., shock, distancing, inappropriate comments) reported significantly higher levels of fear of progression compared to those who received support and understanding. Conclusions: Fear of disease progression may constitute a substantial psychological burden in patients with alveolar echinococcosis. In this sample, FoP was significantly associated with psychological and psychosocial variables, including negative social reactions, whereas no statistically significant associations were detected with the evaluated medical and treatment-related variables. These findings highlight the importance of considering the psychosocial context of AE and support the integration of psychological assessment and support into the clinical care of affected patients.

## 1. Introduction

Echinococcosis is a parasitic disease caused by the larval forms of tapeworms belonging to the genus *Echinococcus*. In humans, infection is most commonly attributed to the *Echinococcus granulosus sensu lato* species complex, which causes cystic echinococcosis, and *Echinococcus multilocularis*, responsible for alveolar echinococcosis (AE) [1]. The life cycle of *E. multilocularis* involves an intermediate host, typically rodents from the subfamily Arvicolinae. Humans become accidental intermediate hosts through the ingestion of parasite eggs present in contaminated food or water [2]. AE cases are reported across the Northern Hemisphere, extending from Western, Northern, and Eastern Europe to Asia, and encompassing regions that span from Eastern Turkey through Central Asia to Western and Northern China. Over recent years, in Europe, AE is emerging in almost every country where it was detected, with two major epicentres in the Alpine and the Baltic areas, including Poland [3].

The liver is the organ most commonly affected by primary lesions in AE [4]. In some patients, distant lesions are already present at the time of diagnosis, involving organs such as the lungs, kidneys, spleen, or brain. If untreated, AE is a progressive, life-threatening disease, but due to advances in the treatment of this parasitic disease, the prognosis in AE patients has improved significantly [5,6]. The treatment of choice for AE is radical surgical resection combined with pharmacological therapy using benzimidazoles, which can typically be discontinued two years after surgery. However, radical surgery is only feasible in 20–50% of patients due to the advanced stage of parasitic disease at the time of diagnosis [7].

In patients for whom radical surgery is not an option, long-term antiparasitic treatment with benzimidazoles is mandatory [7]. Such therapy is highly effective in stabilizing the disease course [6], although most of these individuals do not meet the criteria for temporary suspension of pharmacological therapy over extended periods. Extended benzimidazole therapy is associated with the risk of adverse effects and represents a substantial financial burden for patients in Poland. A systematic literature search revealed evidence of associations between parasitic infections and mental illness in human populations in Africa [8]. Research has shown that individuals with AE experience high levels of fear of disease progression (FoP), with FoP scores reported to exceed those observed in oncology patients. In a study conducted by Nikendei et al. (2019), patients with AE exhibited elevated levels of depression and anxiety symptoms compared to the general population, and their symptoms of post-traumatic stress were comparable to those seen in individuals with cancer [9]. Furthermore, qualitative research by Nikendei et al. (2023) revealed that patients perceive AE as a serious threat that adversely affects their psychological and social well-being [10]. Participants reported concerns regarding uncertainty about the future, the possibility of disease recurrence, and the impact of the illness on their social relationships and daily functioning. The health -related quality of life in AE patients differs from that of the healthy population with respect to mental aspects [11].

The World Health Organization (WHO) [12] underscores the necessity of integrating psychological support into the care of patients with parasitic diseases such as AE. Specifically, WHO recommends integrating mental health assessments, including screening for depression and anxiety, into routine patient care. Furthermore, WHO advises providing patients with information about the disease and effective strategies for coping with stress in order to optimize their psychological well-being.

FoP represents a significant psychological burden for patients diagnosed with AE. Given the often asymptomatic and slow nature of the disease course, many individuals struggle with uncertainty regarding potential future health deterioration. Even after completing treatment, concerns about recurrence or undetected disease may be accompanied by chronic stress and anxiety symptoms. The necessity of long-term medical surveillance, combined with the risk of serious complications, can evoke a persistent sense of threat and profound fear of the unknown.

Furthermore, the psychological impact of this fear may extend beyond the purely medical realm and affect patients’ daily functioning and mental well-being. Individuals with AE may contend with intrusive health-related thoughts, which can result in sleep disturbance, difficulty concentrating, and even avoidance of medical appointments due to apprehension of receiving unfavorable results. Persistent anxiety symptoms may negatively affect quality of life and, in some cases, be associated with broader mental health difficulties. Extensive reviews of research on parasitic diseases, particularly in developing countries, have demonstrated a significant association between parasitic infection and an increased risk of mental health disorders compared to the general population, as reported in a systematic review [8]. Understanding the psychological mechanisms underlying patient experiences with parasitic diseases is thus a prerequisite for designing effective emotional support strategies in clinical practice.

The primary aim of our study was to assess the intensity of FoP among patients diagnosed with alveolar echinococcosis treated with different therapeutic approaches and to identify specific medical and psychosocial factors that were associated with its severity. In contrast to previous studies, the present study specifically examines FoP and its association with social reactions following disclosure of the diagnosis in a Polish AE patient population. This research sought to deepen the understanding of the emotional consequences of chronic parasitic disease and to determine practical directions for psychological support tailored to the care of this patient group.

## 2. Materials and Methods

This was an observational and cross-sectional study, with data collected at a single, specific time point. Participation was strictly voluntary, and anonymity was guaranteed to all patients. The project received approval from the local Independent Bioethics Committee (approval no. NKBBN/496/2022). This article represents a part of a larger research project, the execution of which relied on the material collected during this initiative.

The primary data collection tool used in the study was a custom-designed survey that gathered socio-demographic, psychological, and clinical data pertaining to the disease, its treatment, and the organizational aspects of care. The psychological domain of the questionnaire comprised several items measured using a numerical rating scale (NRS). The NRS was used as a simple measure of the self-reported intensity of fear and stress. Participants used this scale to respond to the following questions:

- Do you experience fear of disease progression (i.e., advancement) and its consequences? (Responses ranged from 0—no fear, to 10—very intense fear).
- How would you rate your level of stress during the diagnostic process? (Responses ranged from 0—no stress—to 10—very intense stress).
- How would you rate your level of stress during the treatment of echinococcosis? (Responses ranged from 0—no stress, to 10—very intense stress).

The final item, developed specifically for this study, invited open-ended responses without any predefined options. It addressed social reactions to the illness and was phrased as follows: “What were/are other people’s reactions to the information about your illness?” Responses to this open-ended question were independently classified by two clinical psychologists as positive/supportive or negative/non-supportive. A third psychologist was designated to resolve any disagreements; however, the two raters reached agreement on all classifications, and third-rater adjudication was therefore not required. 

Two standardized questionnaires were also used in the study:

1. GAD-7 (Generalized Anxiety Disorder-7) is a tool designed to assess the severity of symptoms indicative of generalized anxiety disorder (GAD). The questionnaire was developed by Spitzer, Williams, Kroenke, and Löwe in 2006 [13]. It comprises seven items that quantify the frequency of anxiety symptoms experienced over the preceding two weeks. Responses are provided on a four-point scale ranging from 0 (“not at all”) to 3 (“nearly every day”). The individual items address feelings of tenseness and nervousness, irritability, uncontrollable worrying (including fears that something dreadful might happen), and difficulty relaxing. The questionnaire is well-validated for its robust psychometric properties and is extensively used in both scientific research and clinical settings for screening purposes. The validated Polish-language version of the GAD-7 was used in the present study. The total score was calculated as the sum of the seven items, yielding a score ranging from 0 to 21, with higher scores indicating greater severity of anxiety symptoms.
2. PHQ-9 (Patient Health Questionnaire-9) is a concise self-report instrument used to measure the severity of depressive symptoms. Developed in 2001 by Kroenke, Spitzer, and Williams, the tool consists of nine items, each directly corresponding to the DSM diagnostic criteria for depression [14]. The questions assess the frequency of various symptoms over the past two weeks, including anhedonia, feelings of sadness and hopelessness, sleep disturbance, fatigue, appetite changes, low self-esteem/self-worth, difficulty concentrating/impaired concentration, psychomotor changes, and self-harm thoughts. Responses are scored on a four-point scale, ranging from 0 (“not at all”) to 3 (“nearly every day”). In our study, we additionally included item 10 from the PHQ-9, which assesses the degree to which depressive symptoms have interfered with daily functioning, such as work performance, household responsibilities, and interpersonal relationships. Owing to its strong psychometric properties, simplicity, and alignment with diagnostic criteria, the PHQ-9 is widely adopted for both the diagnosis and monitoring of depression severity. The validated Polish-language version of the PHQ-9 was used in the present study. The PHQ-9 total score was calculated as the sum of the nine symptom items, yielding a score ranging from 0 to 27, with higher scores indicating greater severity of depressive symptoms. The additional item 10 was not included in the PHQ-9 total score and was analyzed separately as a measure of the impact of depressive symptoms on daily functioning.

### 2.1. Statistical Analysis

Statistical analyses were performed using TIBCO Statistica, version 13.3 (TIBCO Software Inc., Warszawa, Poland). Due to the small sample size and the characteristics of the analyzed variables, non-parametric statistical methods were used. Differences between two independent groups were assessed using the Mann–Whitney U test, whereas associations between variables were examined using Spearman’s rank correlation coefficient. All statistical tests were two-tailed, and a *p*-value < 0.05 was considered statistically significant. As data were collected using paper-based questionnaires in routine clinical settings, individual responses were occasionally missing; missing values were not imputed, and analyses were based on available data. Given the exploratory nature of the analyses, no formal adjustment for multiple comparisons was applied. Therefore, the findings, particularly those based on multiple correlation analyses, should be interpreted cautiously and regarded as hypothesis-generating.

### 2.2. Participants 

Patients with a diagnosis of probable or confirmed AE (according to Brunetti et al. [4]), either currently or previously treated for the condition, who were hospitalized at University Centre of Maritime and Tropical Medicine during the one- year study period were recruited. All patients who were offered participation in the study consented. The diagnosis of AE was established based on consistent clinical data, imaging findings, and serological test results, as well as histopathology or PCR tests in some cases. The majority of participants originated from the Warmian-Masurian Voivodeship (48.6%) and the Pomeranian Voivodeship (17.1%), followed by the Subcarpathian (5.7%), Masovian (5.7%), Podlaskie (5.7%), and Silesian (5.7%) regions.

The mean time since diagnosis was nearly 9 years (with a standard deviation of approximately 6 years). Most patients were receiving ongoing antiparasitic treatment. Albendazole was the standard treatment, administered to all patients except for one individual who received mebendazole due to intolerance to albendazole. Notably, over half of the patients (51%) sourced their medication independently from abroad due to the high cost of this drug in Poland. A majority also reported experiencing adverse effects (63%). Regarding surgical history, 8 patients (22.8%) had undergone liver transplantation, 12 patients (34.4%) underwent radical surgery, 3 patients (8.6%) underwent non-radical surgery, and 12 patients (34.3%) were treated exclusively with pharmacotherapy. 

The mean age of the study group was approximately 55 years with a standard deviation of about 13 years. The cohort was composed of 54% women and 46% men, with most participants reporting having two children. The participants represented various educational backgrounds, with a slight predominance of individuals with vocational education. Most were either employed or retired and indicated generally favorable economic circumstances. Detailed characteristics of the study group are provided in Table 1.

## 3. Results 

**Figure 1:**
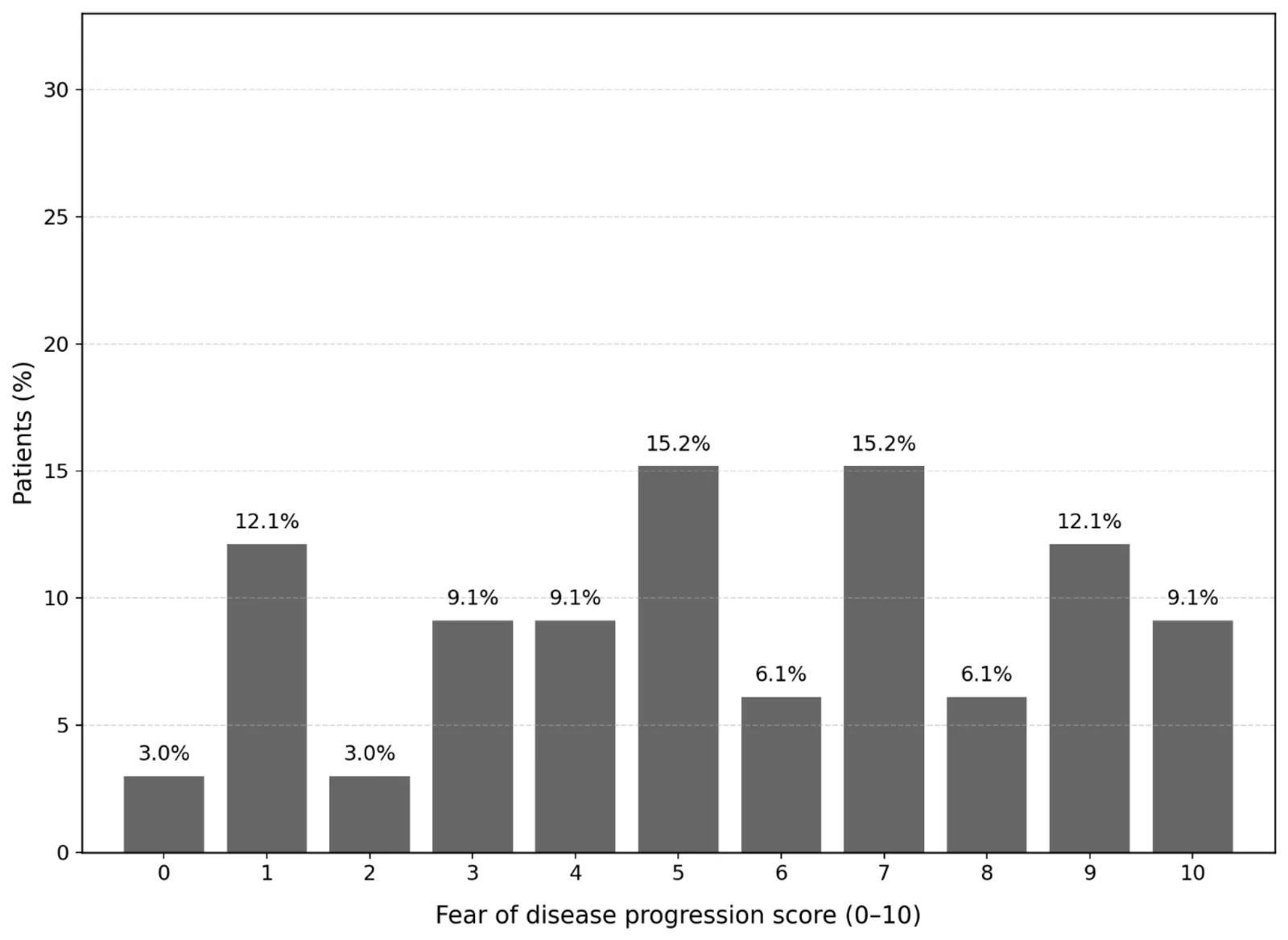
Intensity of fear of disease progression among patients with alveolar echinococcosis.

Patients evaluated their FoP using a Numerical Rating Scale (NRS 0–10). The mean FoP score was 5.48 (SD = 2.98), with a median of 5 (Q1 = 3; Q3 = 8). Overall, the group reported a moderate level of fear related to the progression of AE; however, a considerable variability in responses was observed. Notably, seven patients in the study cohort rated their FoP as maximal or near-maximal (scores of 9 or 10). These findings are presented in Figure 1, which illustrates the distribution of FoP scores across the 0–10 scale among the patients. No statistically significant differences in FoP were observed across medical variables. Specifically, no statistically significant differences in FoP scores were observed between patients receiving and not receiving antiparasitic treatment, between those who imported medication from abroad and those who did not, or between patients experiencing versus not experiencing adverse drug effects. Similarly, there were no statistically significant differences in FoP between patients who had undergone radical surgery or were awaiting surgical intervention, and those for whom such procedures were not feasible. All analyses were performed using the Mann–Whitney U test and are presented in Table 2. 

Table 3 shows the association between FoP and duration of disease, which was also not statistically significant. Neither the duration of disease nor the duration of treatment correlated with patients’ FoP (Spearman’s rho). Table 3 also presents additional, statistically significant correlations with the FoP. Several psychological variables were significantly associated with FoP. FoP was significantly correlated with depressive symptoms and with the extent to which these symptoms interfered with daily functioning. Specifically, greater FoP was associated with a higher severity of depressive symptoms reported by patients. Similarly, higher levels of generalized anxiety and stress experienced during diagnosis and treatment were associated with stronger fear of progression.

Social context was also associated with patients’ FoP. Responses to the open-ended question regarding others’ reactions to the diagnosis were classified by two clinical psychologists as positive/supportive or negative/non-supportive. Patients’ reports of negative reactions included expressions of fear of contagion, shock or surprise, distancing, and inappropriate comments. The Mann–Whitney U test revealed statistically significant differences in FoP between patients who encountered constructive versus non-constructive reactions (Table 4).

**Figure 2:**
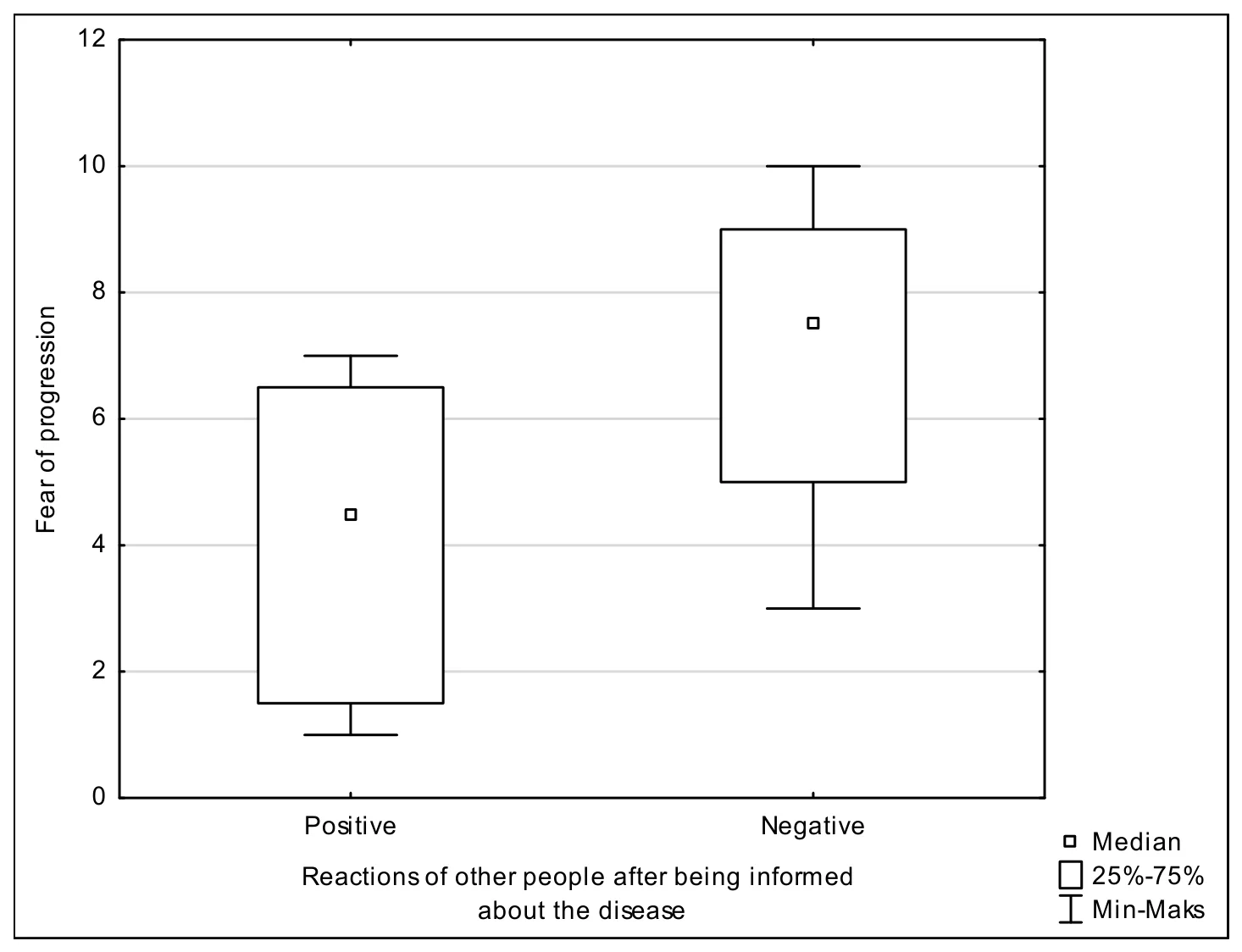
Differences in fear of disease progression between patients who experienced different social reactions after disclosing their diagnosis of alveolar echinococcosis.

This finding is additionally illustrated in Figure 2, which highlights that patients who encountered negative/non-constructive reactions after disclosing their AE diagnosis reported significantly higher levels of FoP compared to those who received supportive and understanding responses.

Particular attention should be paid to the predominance of negative reactions encountered by patients within both their close and broader social circles. Most of the non-constructive responses were associated with expressions of shock, surprise, and disbelief that such a disease even exists. Unfortunately, some patients also reported experiences such as social withdrawal, malicious remarks, and—as described by one female patient—colleagues calling her workplace to question why someone undergoing treatment for AE had returned to work and was now allegedly putting others at risk of infection. In the present study, negative social reactions were associated with higher levels of FoP; however, given the cross-sectional design, the direction of this relationship cannot be determined.

## 4. Discussion

The aim of this study was to assess the intensity of fear of disease progression among patients diagnosed with AE in Poland. AE remains a significant diagnostic and therapeutic challenge in Poland. Based on the 2025 systematic review from the KNOW-PATH project by Adriano Casulli et al. [3], there were 354 documented cases of human alveolar echinococcosis in Poland between 1997 and 2023.

Patients after liver transplantation constituted a relatively large group of patients (22.9%). This is unique, because liver transplantation in AE patients is nowadays a rare treatment, performed mainly for life-threatening indications. These patients undergo regular check-ups at our center due to long- term treatment with albendazole and the risk of disease relapse. Patients after radical surgery, who have completed pharmacological treatment and who have not experienced disease progression after several years of follow-up, usually prefer to undergo check-ups at medical centers in their place of residence. The relatively small number of patients in the study receiving only pharmacological treatment is probably due to the fact that most of them are elderly with multimorbidity. For them, traveling to our center for checkups is a problem, so some of them prefer to stay on long- term treatment with albendazole and remain under the care of their family physician. 

The findings of the study indicate that a substantial proportion of patients experience a high level of FoP, as reflected by the fact that seven individuals in the study group rated this fear as maximal or near-maximal (scores of 9 or 10 on a ten-point scale). This observation is consistent with previous reports by Nikendei et al. (2019), who found that patients with AE exhibited elevated levels of anxiety and depression, with FoP exceeding that observed even among oncology patients [9]. 

The study did not reveal any statistically significant associations between medical variables (such as pharmacological treatment, its source, experience of adverse effects, or qualification for radical surgical intervention) and the intensity of FoP. In contrast, significant associations were observed between FoP and several psychological and psychosocial variables. Similar conclusions were drawn by Nikendei et al. (2023), who, in a qualitative study, demonstrated that patients often perceive the disease as threatening and difficult to comprehend, regardless of its actual stage of advancement [10].

The nature of social reactions following disclosure of the diagnosis was significantly associated with FoP levels. Patients who experienced non-constructive, negative responses from others—such as shock, surprise, malicious remarks, or even exclusionary behavior in the workplace—reported significantly higher FoP levels compared to those who received support and understanding. This finding aligns with observations published by the World Health Organization (2020), which emphasize that social stigmatization and lack of emotional support can contribute to deterioration in the psychological well-being of patients with parasitic diseases [12].

These social reactions point to a serious issue related to low public awareness of AE. The surprise expressed by others upon learning that such a disease exists, together with fears of contagion, may contribute to experiences of alienation and social stigmatization. In the present study, negative social reactions were associated with higher FoP levels; however, the direction of this relationship cannot be established. Similar mechanisms have been documented in the literature on other stigmatizing diseases, such as HIV, where lack of public knowledge results in prejudice and social isolation of affected individuals [15]. Low public awareness of AE is not limited to isolated experiences; it has been confirmed by studies across Europe, which revealed substantial differences in knowledge about *Echinococcus multilocularis* between countries, as well as a generally low sense of being well-informed, even in highly endemic regions [16]. The authors of these studies emphasize the need for targeted educational programs to help the public realistically assess the risks and reduce unnecessary fear and stigmatization of patients. In light of these findings, it can be concluded that the lack of reliable information about AE in the public sphere not only hampers prevention efforts but also contributes to social misunderstanding, which may negatively affect patients’ psychological well-being.

To summarize, the findings underscore the importance of addressing the psychosocial context in the management of AE. In this sample, FoP was associated with psychosocial variables, including social reactions, whereas no statistically significant associations were detected with the evaluated medical variables. Therefore, it is essential to provide patients not only with comprehensive medical care, but also with access to psychological support and public education initiatives aimed at fostering awareness and reducing stigma.

From a clinical perspective, these findings support the inclusion of psychological assessment and support in the care of patients with AE. Particular attention should be given to FoP, anxiety and depressive symptoms, as well as to patients’ social experiences related to the disclosure of their diagnosis. The assessment of anxiety symptoms and FoP using appropriate validated psychometric instruments should be considered [17], together with access to psychological support during diagnosis, treatment, and follow-up. Educational initiatives aimed at increasing public awareness of AE and reducing stigmatizing social reactions may also be warranted, particularly given the association between negative social reactions and higher FoP observed in the present study and previous findings concerning stigmatizing diseases [15]. FoP may also be considered within interdisciplinary care, particularly given the chronic course of AE and uncertainty regarding prognosis.

Several limitations of this study should be acknowledged. The relatively small sample size and single-center recruitment limit the generalizability of the findings and may have reduced the statistical power to detect associations. The characteristics of the sample, including the relatively high proportion of liver-transplanted patients and possible underrepresentation of older patients receiving exclusively pharmacological treatment, may also have introduced selection bias. The cross-sectional design precludes causal conclusions. FoP was assessed using a single-item NRS rather than a validated multidimensional FoP questionnaire, limiting direct comparisons with studies using validated measures. In addition, retrospective ratings of stress during diagnosis and treatment may have been affected by recall bias, particularly given the long duration of disease, and their associations with current FoP should therefore be interpreted cautiously. Finally, missing responses resulted in varying numbers of valid observations across analyses; missing data were not imputed. Despite these limitations, to our knowledge, this is the first study conducted in Poland to specifically examine fear of disease progression and its psychosocial correlates in patients with AE, providing initial data on this previously unexplored aspect of AE in the Polish patient population.

## 5. Conclusions 

Fear of disease progression represents a substantial psychological burden in patients with AE. In this sample, no statistically significant associations were detected between FoP and the evaluated medical and treatment-related variables, whereas significant associations were observed with several psychological and psychosocial variables. From a clinical standpoint, it is essential to implement systemic measures that support not only effective antiparasitic treatment but also the mental health of patients. These findings highlight the importance of considering the psychosocial context of AE in the clinical care of affected patients.

## Figures and Tables

**Table 1:** Sociodemographic and clinical characteristics of the study group.

Variable (*n* = 35)	*n* (%) or M (SD)
Age, years	55.15 (12.59)
Sex	
Women	19 (54.3%)
Men	16 (45.7%)
Education	
Higher	7 (21.2%)
Secondary	8 (24.2%)
Vocational	13 (39.4%)
Primary	5 (15.2%)
Item nonresponse	2 (5.7%)
Employment status	
Employed	12 (34.3%)
Retired	13 (37.1%)
Disability pension	9 (25.7%)
Unemployed	1 (2.9%)
Self-reported financial situation	
Very good	4 (11.4%)
Good	17 (48.6%)
Average	13 (37.1%)
Poor	1 (2.9%)
Number of children	
None	8 (23.5%)
One	7 (20.6%)
Two	13 (38.2%)
Three	4 (11.8%)
Four or more	2 (5.9%)
Item nonresponse	1(2.9%)
Disease duration, years	8.90 (6.30)
Current antiparasitic treatment	
Yes	23 (74.2%)
No	8 (25.8%)
Item nonresponse	4(11.4%)
Independent sourcing of medication	
Yes	18 (62.1%)
No	11 (37.9%)
Item nonresponse	6(17.1%)
Adverse effects of antiparasitic treatment	
Yes	22 (68.8%)
No	10 (31.3%)
Item nonresponse	3(8.6%)
Surgical treatment history	
Liver transplantation	8 (22.9%)
Radical surgery	12 (34.3%)
Non-radical surgery	3 (8.6%)
Pharmacotherapy only	12(34.3%)
Currently qualified for surgery	1(2.9%)

Note. *n* = number of participants; M = mean; SD = standard deviation. Percentages were calculated based on the number of valid (non-missing) responses for each variable. Missing data (item nonresponse) were not imputed and are marked accordingly in the table; frequencies of missing responses per variable are reported in.

**Table 2:** Differences in fear of disease progression according to selected medical and treatment-related variables using the Mann–Whitney U test.

Variable	Yes: *n*, Median	No: *n*, Median	*p*	Effect Size r
Current antiparasitic treatment	*n* = 23, Mdn = 5	*n* = 8, Mdn = 5	0.318	0.18
Independent sourcing of medication	*n* = 18, Mdn = 6	*n* = 11, Mdn = 5	0.928	0.02
Adverse effects	*n* = 22, Mdn = 5	*n* = 10, Mdn = 7	0.244	0.20
Surgery performed/qualified vs. not eligible	*n* = 17, Mdn = 6	*n* = 6, Mdn = 7	0.597	0.11

Note. *n* = number of participants; Mdn = median; *p* = *p*-value; r = effect size. Analyses were conducted using valid (non-missing) responses for each variable; missing data were not imputed.

**Table 3:** Associations between fear of disease progression and disease-related and psychological variables.

Variable	Spearman’s Rho	*p*-Value
Disease duration	−0.27	0.152
Depressive symptoms (PHQ-9)	0.47	0.006
Functional impairment due to depressive symptoms (PHQ-9, item 10)	0.40	0.029
Generalized anxiety symptoms (GAD-7)	0.42	0.016
Stress experienced during diagnosis	0.62	0.001
Stress experienced during treatment	0.69	0.001

Note. Spearman’s rank correlation coefficients are reported. Given the exploratory nature of the analyses, no adjustment for multiple comparisons was applied.

**Table 4:** Fear of disease progression according to reported social reactions using the Mann–Whitney U test.

Variable	Positive/Supportive Reactions	Negative/Non-Supportive Reactions	*p*	Effect Size r
Fear of disease progression	*n* = 8, Mdn = 4.5	*n* = 18, Mdn = 7.5	0.015	0.48

Note. *n* = number of participants; Mdn = median; r = effect size. Twenty-six participants provided responses to the open-ended question regarding social reactions and were included in this analysis (positive/supportive, *n*=8; negative/non-supportive, *n* = 18). Nine participants did not provide a response to this item and were therefore not included in the analysis.

## Data Availability

The study data are available from the author upon request.
